# Report on the Health of the London Postmen in 1856

**Published:** 1857-04

**Authors:** Waller Lewis

**Affiliations:** Medical Officer to H.M. Post-Office.


### REPORT ON THE HEALTH OF THE LONDON POSTMEN IN 1856.

By WALLER LEWIS, M.B.Cantab., Medical Officer to H.M. Post-Office.

[The following Report has, with the kind permission of his Grace the Duke of Argyll, been forwarded to us by our able collaborateur Dr. Waller Lewis. We are deeply indebted, and hope that in time a similar report on the sanitary condition of all the Government departments will be annually added to the literature of the country. Such reports are invaluable in science and in history. EDITOR.]

Medical Department, General Post Office, Feb. 1857.

SIR,—I have now the honour of presenting my second report on the health of the officers of this department.

NUMBER OF OFFICERS UNDER MEDICAL CHARGE..—By the regulations now in force the various officers may be arranged, as to their relation to the medical officer, in three classes, according to their positions and salaries.

*Class A.*—On those whose salaries exceed £150 a year the medical officer does not attend, except during the prevalence of an epidemic, or in cases of sudden illness or accident at the office.

*Class B.*—Clerks whose salaries do not exceed £150 per annum are not supplied with medicines.

*Class C.*—All other officers employed and paid by this department, who are attached to the chief, or Money-order Office, including inspectors, sorters, sub-sorters, letter-carriers, messengers, porters, labourers, and domestics (male and female), are prescribed for at the office, or visited at their own houses when too ill to attend. Medicines and instruments are also gratuitously supplied to them.

From various causes the number of officers in what is called "the minor establishment, or class C," is constantly varying. Some of the lately appointed letter-carriers, on presenting themselves for re-examination to the medical officer at the expiration of their six months' probation, are found to be unfit for the duties, and are, therefore, not confirmed. Some, who are disabled from attending their duties by continued illness, are temporarily replaced by substitutes; whilst interchanges between the chief and branch offices are constantly taking place. For these reasons, many more officers are under medical care, in the course of the whole year, than are entered on the books, as attached to the office at any one time.

Thus, on the 31st December, there were—

| | |
|---|---|
| In class A - - - - - - | 274 |
| In class B - - - - - - | 331 |
| In class C - - - - - - | 1,100 |

making 1,431 in the two last classes ; while, during the course of the year, there have been 1,969 different individuals attached, for a longer or shorter period, to the two offices, any of whom, if ill, might have applied for official medical aid. As a considerable proportion of the officers constituting the difference between these two numbers have helped to swell the invalid list, classes B and C may be stated as averaging 1,500 for the entire year.

CANDIDATES EXAMINED.—During the year 401 candidates have obtained nominations as clerks, letter-carriers, etc., and, in accordance with the regulations, have presented themselves to me for medical examination. Of this number forty-seven, or nearly 12 per cent., were rejected, and 354 passed satisfactorily.

Of this latter number, sixty-one, or about 16 per cent., were subsequently rejected by the Civil Service Commissioners, making 108 rejections among 401 candidates.

GENERAL HEALTH.—The health of the whole body of officers has been highly satisfactory. It is most gratifying to me to be able to state that, among the 605 officers, in classes A and B, there is not a single death to report.

Most of the illness that has taken place in these classes has been of a slight character. I am inclined to attribute much of this satisfactory condition to the annual monthly holiday given to all these officers, which is mostly spent in the country.

TABULAR STATEMENT

*Of the Number of Clerks and other Officers who presented themselves for Medical Treatment during the year* 1856, *specifying some of the more common complaints.*

| Months. | Clerks. | Other officers. | Total No. of officers. | Diarrh. | Rheumatism. | Sore throat. | Boils. |
|---|---|---|---|---|---|---|---|
| January ...... | 9 | 130 | 139 | 10 | 13 | 2 | 3 |
| February ...... | 11 | 105 | 116 | 5 | 8 | .. | 3 |
| March ......... | 12 | 118 | 130 | 3 | 13 | 3 | 5 |
| April.......... | 16 | 107 | 123 | 9 | 19 | 2 | 3 |
| May .......... | 5 | 140 | 145 | 7 | 14 | 12 | 2 |
| June.......... | 16 | 128 | 144 | 20 | 14 | 14 | 10 |
| July .......... | 16 | 173 | 189 | 52 | 10 | 6 | 2 |
| August........ | 31 | 214 | 245 | 102 | 4 | 4 | 5 |
| September .... | 9 | 149 | 158 | 25 | 6 | 3 | 6 |
| October ...... | 8 | 97 | 105 | 8 | 7 | 4 | 5 |
| November .... | 12 | 119 | 131 | 8 | 10 | 8 | 3 |
| December .... | 14 | 132 | 146 | 4 | 10 | 6 | 4 |
| Total ...... | 159 | 1612 | 1771 | 258 | 128 | 64 | 51 |

About 650 visits have been paid to officers at their own houses, when too ill to attend at the office.

It will be seen by the above table that, during the year, the large number of 1,771 distinct cases of illness have presented themselves for treatment. The total number of officers having permission to avail themselves of my care being estimated at 1,500, it follows that, on the average, each officer has suffered from illness rather more than once. By far the larger number of these cases were of little gravity, as I have always encouraged the men to present themselves for advice on the first symptoms of illness shewing themselves. In the majority of the cases thus early attended to, the disease has been nipped in the bud, and a lengthened illness, with consequent absence from duty, prevented. This, I believe to be one of the most efficient means for practically carrying out the science of preventive medicine. It is in this way that the officers themselves derive the most important advantage from the liberality of the Government in providing gratuitous medical advice, as suggested by the late Postmaster-General. Not only are they saved the cost of medical attendance, but much absence from duty, with the consequent loss of a proportion of their salaries, is by this means spared them; they are also spared the bodily and mental pain that accompanies, and the injury to the constitution which follows, a severe and prolonged attack of illness.

In the table I have specified a few of the diseases more commonly met with among the officers—namely, diarrhœa, rheumatism, inflammatory sore throat, and boils or carbunculoid disease. These are all, to a certain degree, more or less preventible. They are mostly due to impure air, or exposure to draughts, or to sudden and great changes of temperature, or to improper diet, or insufficient clothing. Other ailments, which I am occasionally called on to prescribe for, I have traced to neglect of personal cleanliness. In accordance with a report that I made on this subject in the course of the summer, the authorities have directed that more attention should, in future, be paid to this subject as regards letter carriers. It will be for the benefit of the men themselves, that the regulation alluded to shall be strictly enforced.

Epidemics.—There have been no special epidemics during the year; but the usual summer diarrhœa has been more than usually prevalent. The table shews that 154 officers suffering from that complaint presented themselves for treatment during the months of July and August alone. But these figures do not represent the actual number attacked, as many more were relieved without personal application to the medical officer.

During the summer season, sixteen of the offices in this building and the Money-order Office were kept constantly supplied with the proper remedies for this complaint, in order that relief might be obtained at any hour of the day or night, immediately on an attack coming on.

Besides the diarrhœa medicine so supplied to the two principal offices, upwards of a hundred gallons were sent to forty branch and district offices in London and the suburbs, for the use of the officers employed there. That much benefit is obtained by this means is clear, from the following letter that has been received, with others, from the charge-taker of one of the branch offices:—

"Stepney Branch, 31 August.

"Sir,—I beg to inform you that we require a fresh supply of diarrhœa medicine at this branch. The former has proved very beneficial—it has been taken in time, and has thereby prevented more serious consequences.—I am, sir, your obedient servant,

"Wm. Bokenham, Esq. "T. Reynolds."

Inflammatory sore throat has been very prevalent. It is traceable, in a large proportion of cases, to draughts of cold air entering through open doors while the men are engaged in sorting and stamping in the larger offices. Many cases of rheumatism arise from the same cause—one of the most difficult evils to deal with in this large building.

There have been fifty-one cases of boils—a large number. I believe this disease to take its origin partly from improper food, and partly from living in an impure atmosphere. It is certainly contagious. I have observed it to be more frequent among the men who live in small, close rooms in the interior of the town, than those who inhabit more open dwellings in the suburbs. In my last annual report I alluded to the great advantages that would accrue to the men if they could be provided with more healthy dwellings. I should expect to see a considerable diminution in the last mentioned disease among the inmates of such improved habitations.

There have been 1,612 cases of sickness among the 1,638 persons composing the minor establishment: of these cases 716 have been severe enough to incapacitate the invalids for a time from duty.

Average Duration of Illness.—*Minor Establishment.*—The total amount (in days) of absence from duty, on account of illness, in class C, was 11,934 days. This will give a mean average duration of 16.66 days for each officer who absented himself. But this amount of absence is unduly swollen by the prolonged absence of four officers, three of whom have not been on duty any part of the year. As none of these will return to the department, and their places have been long filled up by fresh appointments, their absence should be deducted from the gross amount: when this is done, the average duration of each case of illness will be reduced to 15.13 days.

But a large proportion of this reduced amount of absence is caused by comparatively a very few cases. Seven officers have been absent many months consecutively; one for eleven, one for nine, and another for eight months. These seven cases have consumed between them 1,196 days, so that if allowance be made for these cases of prolonged illness, each of which was above three months duration, the average absence from duty of the remaining cases amounts to 13.59 days. This number represents correctly the average duration of each case of illness sufficiently severe to necessitate absence from duty; but if the total amount of absence be divided among all the cases of sickness, it will give an average of 6.69 days absence for each patient.

*Clerks.*—One hundred and fifty-nine clerks have presented themselves for treatment during the year. This number includes a few in class A, who suffered from the prevailing diarrhœa. The total number of heads of departments, clerks, etc., comprehended in classes A and B, who have been off duty, is 254, and the total amount of such absences is 4,939 days—making an average of 19.44 days each.

If fifteen of these cases, however, which have each exceeded three months in duration and have engrossed 2,409 of the whole number of days, be deducted from the gross amount, the mean average duration of each case will be reduced to 10.6 days.

Of the 2,243 officers who have been attached to the Chief and Money-order Offices during some portion of the year, 970 have been at some time absent from duty on account of illness.

The average number of days that any officer was off duty from illness, including in the calculation the whole body of officers, was 7.52 days; but if correction be made of the four exceptional cases first alluded to, the average absence will be exactly seven days;

*Deaths in the Class of Letter-Carriers, etc.*

| Disease. | Age at death. | | | | |
|---|---|---|---|---|---|
| | Under 30. | Under 40. | Under 50. | Under 60. | Total. |
| Consumption ....... | 2 | 1 | 4 | 1 | 8 |
| Heart-disease ........ | .. | 1 | .. | .. | 1 |
| Inflammation of lungs | 1 | .. | .. | .. | 1 |
| Dropsy.... ......... | .. | 1 | .. | .. | 1 |
| Total .......... | 3 | 3 | 4 | 1 | 11 |

making the mortality 7.4 per 1,000—70 per cent. of which was caused by consumption.

Deaths in the Class of Clerks, Heads of Departments, etc.—None.

This favourable statement of the mortality of the whole body of officers is, I believe, partly due to the mildness of the present winter, so far as it has yet gone.

Removable Causes of Disease.—In the previous part of this report, allusion has been made to various complaints to which some of the officers, more particularly the sorters, are unduly subject. Attacks of sore throat, coughs, colds, and rheumatism are frequently attributed to draughts in the larger offices. Where men are engaged in sorting letters or newspapers, directly in front of a door which is being constantly opened, protection has been afforded by means of wicker screens, but these only protect the individuals most directly exposed.

The passages on the secretary's side are complained of during the winter season, as being extremely cold and draughty. I have recommended a fire-place or stove for warming the air that ascends the passages in question, to be erected at the bottom of the grand staircase.

Scarcely a week passes without complaints being made to me of the bad effects on the general health, or on the eye-sight, of the sorters or other officers employed in the larger offices lighted by gas. I have long been of opinion that the method of lighting these and other large offices is capable of very considerable improvement. The lights are very numerous, and being only eight feet from the floor, there is no attempt made to carry off the great amount of caloric, or the deleterious gases given off in combustion.

The number of officers employed at one time in some of the offices, makes it desirable that some better system of combining more perfect lighting, with good ventilation, should be adopted.

In the Inland Office there are employed, at one time on morning duty, about 467 men ; on evening duty, 378 men. The number of gas lamps is 155.

In the newspaper office there are employed at the same time on morning duty, 646 men ; on evening duty, 287—gas burners, 145.

The rapid vitiation of the atmosphere in these offices when lighted up may easily be imagined, when it is recollected that each gas burner gives off as much carbonic acid gas as six human beings, besides the sulphurous acid and other impurities. The temperature in some of the offices during the summer was as high as 86° Fahr.

In a special report on the lighting of the larger offices of this department which I presented to you in December last, I pointed out, at the request of the First Commissioner of Works, the principles that, in my opinion, should be kept in view in lighting rooms or offices where large numbers of persons are collected together. They are the following :—

1. The sources of the light should be as few in number as possible, consistently with the light being sufficiently powerful and equally diffused.

2. The lights should be as elevated as the ceiling will allow.

3. They should be so contrived, that nearly all the heat, as well as the whole products of combustion should be carried off directly, without admixture with the lower strata of air, and be made subservient to an efficient system of ventilation.

4. I am of opinion that the reflective power of metal or other substances might, with economy, be more scientifically employed than is now generally done.

If these principles were carried out in lighting the offices of this department, I believe that very great benefit to the health of a large number of the officers would rapidly result.

Ventilation of the Buildings.—About the end of the year 1855, finding that the various offices situated on the basement floor were in a very unsatisfactory condition with regard to ventilation and aëration, I proposed the laying down a series of tubes for carrying off some of the vitiated air given off by the gas-burners, etc. Considerable benefit was derived from this. A great deal of the dust, so much complained of in the bag-rooms, was carried off through the pipes to the flue of the steam engine, instead of being inhaled by the men.

The walls and ceilings of the bag-rooms and kitchens are no longer blackened, in the course of a few weeks, by the carbon of the imperfectly burned gas. This had long been a source of great mischief to the health of the officers; inducing asthma, bronchitis, and other pulmonary affections. That the officers themselves are sensible of the improvement caused by these measures, is evident from the following communication :—

"Circulation Department, June 21st, 1856.

"Sir,—The bag-room officers, feeling sensible that a considerable improvement has been effected by the system of ventilation lately introduced in the bag department, have requested me to convey to you this acknowledgment, and to thank you on their behalf. Respectfully assuring you, sir, of the pleasure I feel in rendering this testimony to the success which has attended your efforts to promote our health and comfort, I have the honour to be, sir, your humble and obedient servant,

"Dr. Waller Lewis. "Wm. Wiles."

But, although a certain amount of relief has been experienced by this partial ventilation, much more is required to be done before the vaults can be pronounced to be in a healthy condition. When the present building was erected, the business of the office was not to be compared with what it has since become, particularly since the uniform low system of postage.

The basement offices, now used as bag-rooms, kitchens, store-rooms, etc., were not intended for habitation, and were consequently not provided with the requirements and conveniences necessary for health and comfort. Fresh air finds its way, with great difficulty, to many of the offices, and to none of them in sufficient quantity. Vaults, cellars, and subterraneous dwellings, even with the best possible modern appliances, are always more injurious to health than rooms on a level with, or above the surrounding soil, and require more than ordinary care and attention to their due aëration and ventilation.

I have conferred with Mr. Cowper, C.E., on the subject of a more systematic and continuous supply of air to the basement offices, and I agree with him in the belief that the steam engine, at present in use, may be advantageously and economically employed for this object.

Old Cavendish Street Branch Office.—Some months ago I reported on the imperfectly ventilated state of this branch. Originally a private dwelling-house, with very low rooms, it was but ill adapted to accommodate the large number of officers who work there. The recommendations I made for the improvement of that office have been promptly carried into effect, to the great satisfaction and benefit of the men.

I am, Sir, your obedient servant,

Waller Lewis, F.G.S., etc.

To Rowland Hill, Esq., Secretary G. P. O.

---

